# Sequences of C*μ* and the V_H_1 Family in LG7, a Clonable Strain of *Xenopus*, Homozygous for the Immunoglobulin Loci

**DOI:** 10.1155/1992/74187

**Published:** 1992

**Authors:** Melanie Wilson, Anne Marcuz, Michéle Courtet, Louis Du Pasquier

**Affiliations:** Basel Institute for Immunology Grenzacherstrasse 487 Basel CH-3058 Switzerland

**Keywords:** Isogenic *Xenopus*, immunoglobulin, genes

## Abstract

Twenty-eight heavy-chain variable (V_H_1) region genes and the immunoglobulin (IgM) heavy-chain constant region of an isogenic *Xenopus* hybrid, X. *laevis*/X, *gilli*, LG7, have been sequenced. The LG7 clone represents the first *Xenopus* hybrid that is homozygous for the IgH locus. The V_H_1 family was specifically investigated because V_H_1 genes are used by the antibodies produced during the *Xenopus* antidinitrophenol (DNP) response, These V_H_1 germ-line sequences establish a so-called ”dictionary— that is available for studying somatic hypermutational mechanisms in immunized frogs.


*Developmental Immunology*, 1992, Vol. 3, pp. 13–24
Reprints available directly from the publisher
Photocopying permitted by license only

© 1992 Harwood Academic Publishers GmbH
Printed in the United States of America

### Sequences of C$\mu$ and the $V_H1$ Family in LG7, a Clonable Strain of *Xenopus*, Homozygous for the Immunoglobulin Loci

MELANIE WILSON,* ANNE MARCUZ, MICHÈLE COURTET, and LOUIS DU PASQUIER

*Basel Institute for Immunology, Grenzacherstrasse 487, CH-3058 Basel, Switzerland*

Twenty-eight heavy-chain variable ($V_H1$) region genes and the immunoglobulin (IgM) heavy-chain constant region of an isogenic *Xenopus* hybrid, *X. laevis*/*X. gilli*, LG7, have been sequenced. The LG7 clone represents the first *Xenopus* hybrid that is homozygous for the IgH locus. The $V_H1$ family was specifically investigated because $V_H1$ genes are used by the antibodies produced during the *Xenopus* antidinitrophenol (DNP) response, These $V_H1$ germ-line sequences establish a so-called "dictionary" that is available for studying somatic hypermutational mechanisms in immunized frogs.

KEYWORDS: Isogenic *Xenopus*, immunoglobulin, genes.

### INTRODUCTION

The gynogenetic development of diploid eggs produced by *X. laevis*/*X. gilli* hybrids (LG) leads to the production of clones of heterozygous isogenic females (Kobel and Du Pasquier, 1975). The development of the different hybrids and the ease with which large numbers of them can be produced has been a major advantage in the studies of various aspects of the *Xenopus* immune system, particularly antibody diversity (Wabl and Du Pasquier, 1976; Du Pasquier and Wabl, 1978). As useful as they are, these clonable animals are still heterozygous and therefore genetically complex. It would considerably simplify immunogenetic studies if some LG hybrid clones could be made homozygous at the major histocompatibility complex (MHC) or immunoglobulin (Ig) loci. We report here such a clone, LG7, that is homozygous at the Ig heavy-chain (H) locus. In addition, we report most of its $V_H1$ germ-line sequences as well as the sequence of its IgM ($\mu$) heavy-chain gene. This $V_H$ family is involved in the antidinitrophenol (DNP) response, one of the best studied *Xenopus* antibody responses (Brandt et al., 1980; Schwager et al., 1988). Thus, these germ-line sequences establish a "dictionary" necessary for future studies on somatic generation of the antibody repertoire in this species.

*Corresponding author.

### RESULTS

### LG7 Is Homozygous for the Ig Loci

LG7 frogs first attracted our interest because they yielded fewer restriction fragments than other LG hybrids (Schwager et al., 1988). Whether this was due to homozygosity or to a Ig H chromosome loss was not known. To determine the cause of LG7's unique restriction fragment length polymorphism (RFLP) pattern, we decided to first use a variety of techniques. The previous RFLP analyses were confirmed by testing different digests of LG genomic DNAs with probes from all 11 $V_H$ families, light-chain probes for V$\rho$ and C$\rho$ (kindly provided by our colleague J. Schwager; Schwager et al., 1991) and C$\mu$ probes. The individual patterns could be grouped into four RFLP classes, identical for each probe (LG3,15; LG17,5,46; LG14,6; and LG7). LG7 consistently gave half as many restriction fragments as the other LG7 hybrids and Fig. 1 shows examples of Southern blot hybridized with

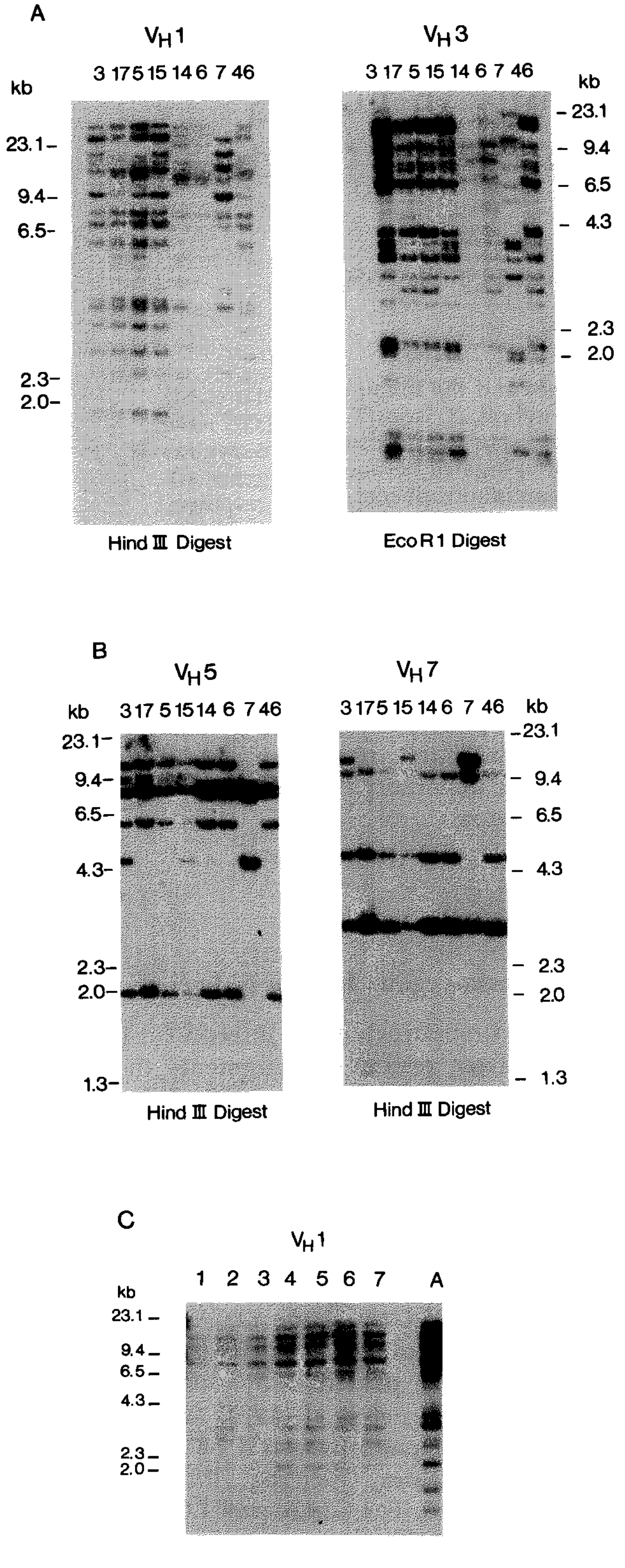

TABLE 1

Absorbance at 405 nm[a]

| Mab specificity | Outbred *laevis* | LG15 | LG7 |
|---|---|---|---|
| 14G1 anti-*laevis* IgM | | | |
| undiluted | 0.38 | 0.13 | 0 |
| 1/5 | 0.46 | 0.17 | 0 |
| 1/50 | 0.29 | 0.09 | 0 |
| 10A9 anti-*Xenopus* IgM | | | |
| undilted | 0.65 | 0.35 | 0.33 |
| 1/5 | 0.86 | 0.31 | 0.27 |
| 1/50 | 0.80 | 0.33 | 0.32 |
| 409B8 anti-*Xenopus* IgL | | | |
| undiluted | 0.11 | 0.14 | 0.10 |
| 1/5 | 0.13 | 0.15 | 0.14 |
| 1/50 | 0.14 | 0.15 | 0.13 |
| 11D5 anti-*Xenopus* IgY | | | |
| undiluted | 0.10 | 0.15 | 0.12 |
| 1/5 | 0.01 | 0.03 | 0.01 |
| 1/50 | 0.04 | 0.04 | 0.02 |

[a]All assays were performed in duplicate and included enzyme-linked antibody controls and substrate controls. Values are results from one data set after 2 hr incubation. No color development occurred in the LG7 wells even after 18 hr; abosrbance ≤0.08.

various $V_H$ probes. Moreover, every band in LG7 that hybridized to a $V_H$ probe could also be found in LG15; thus, LG15 seems to contain an LG7-like haplotype.

To determine whether there are differences in expressed Ig haplotypes, we analyzed the serum from LG7, LG15, and outbred frogs in enzyme-linked immunosorbent assays (ELISA) with different anti-*Xenopus* Ig monoclonal antibodies (Mab). LG7 was always negative with Mab 14G1, a monoclonal that detects specifically *laevis* $\mu$ Ig (Du Pasquier et al., 1985), indicating that only *gilli* $\mu$-type Ig is produced. All three frogs routinely tested positive with Mabs for *Xenopus* IgY (Mab 11D5), *Xenopus* light chain (Mab 409B8), and Lg $\mu$ (Mab 10A9; Table 1). Additional evidence for LG7 possessing only the *gilli* Ig haplotype was also found by screening LG7 genomic libraries with a C$\mu$ probe. Only *gilli* $\mu$ constant

FIGURE 1. Genomic Southern blot analyses of erythrocyte DNA from LG frogs. DNA was digested to completion with either Hind III or EcoRI and electrophoresed on 0.7% agarose gels and Southern blotted. The filters were hybridized with probes for (A) $V_HI$, $V_HIII$, (B) $V_HV$, $V_HVII$, and washed under stringent conditions. Numbers correspond to LG nomenclature (Kobel and Du Pasquier, 1977). (C) Southern blot analysis of LG7 small-egg progeny. Genomic DNA from seven individual LG7 small-egg tadpoles (numbered 1-7) and an adult LG7 (labeled A) were digested with EcoR1 and hybridized with a $V_H$ probe. Markers (in kb) are indicated in the margins.

regions could be sequenced from two different LG7 genomic libraries. Even though *laevis* and *gilli* $C\mu$ cross-hybridize and share long stretches of sequence identity, the introns are of different sizes, with characteristic repeats and thus are easy to identify (Du Pasquier, unpublished).

Although the foregoing data did not exclude loss of a chromosome or deletion of one of the Ig loci, additional experiments do so. Chromosome spreads of phytohemagglutinin-stimulated LG7 spleen cells contained 36 chromosomes, the number expected of LG hybrids. Moreover, when Southern blots were titrated with $C\mu$ and $C\rho$ probes, the signal intensity of LG7 genomic DNA was equal to that of LG15 and LG14, that is, LG7 contains the same number of copies of $C\mu$ and $C\rho$ as LG15 and LG14 (data not shown).

LG hybrids produce two types of eggs, small eggs that can be either normal haploids or aneuploids, and large genetically identical diploid eggs that are used to propagate the species. If LG7 was heterozygous, segregation of IgH (or any other gene family) polymorphism should be detected in progeny. However, the segregation cannot be followed in the large (2n) eggs because they are always identical. Instead, the small eggs (1n) that contain a segregated set of chromosomes must be analyzed. We compared in Southern blots digests of genomic haploid LG7 embryo DNA with adult LG7 DNA. The RFLP pattern of every haploid embryo tested was identical with the adult parent LG7 pattern when tested with different $V_H$ probes and with a $C\mu$ probe (Fig. 1C). Thus, LG7 is homozygous at the IgH locus. This and the fact that the LG7 pattern of Ig H-chain genes is identical to half of the pattern of LG15 Ig genes suggests that LG7 is homozygous for at least the *gilli* Ig H-chain gene set present in LG15.

### LG7 Is Derived from the Gynogenetic Development of an LG15 Small Egg

Small eggs rarely develop beyond hatching unless the second polar body is not extruded so that diploidy is restored and a viable embryo is created. This is a rare occurrence. When small egg embryos do survive, their genetic loci will still be heterozygous if there is a crossover between a locus and the centromere. Thus, heterozygosity is a function of gene position; heterozygosity increases with increasing distance from the centromere (Nace et al., 1970; Kobel and Du Pasquier, 1977).

We believe that the LG7 hybrid developed from an accidentally selected viable diploid LG15 small egg (see Fig. 2). Because all LG hybrids are tested by skin graft as a routine control, this

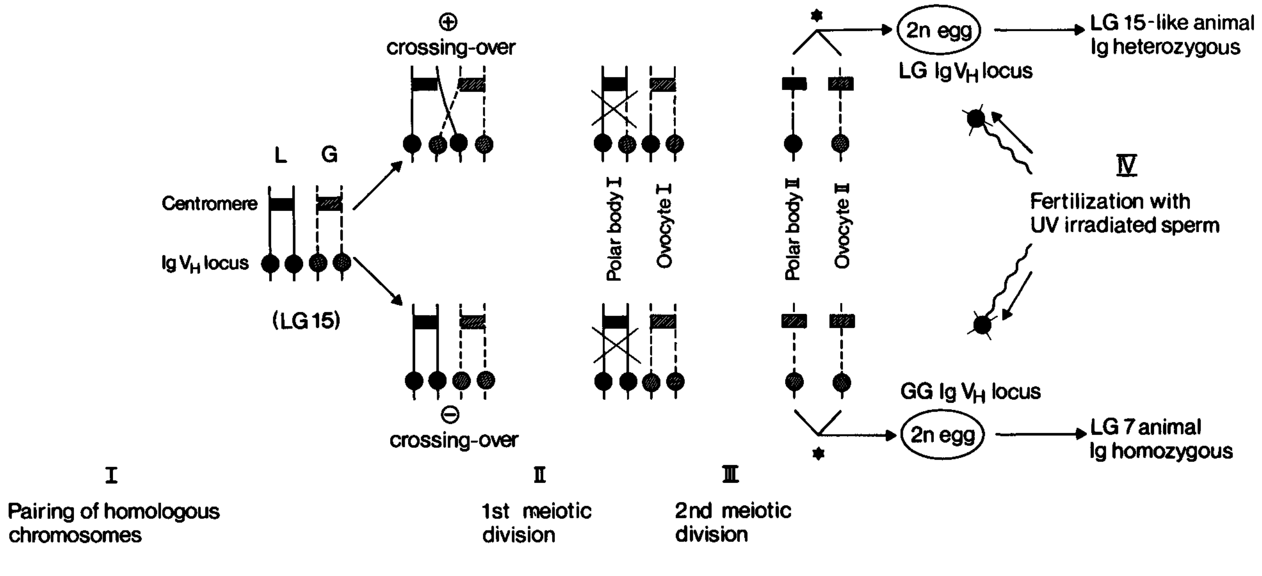

FIGURE 2. Scheme depicting LG7 origins. Pairing of *laevis* (solid lines) and *gilli* (hatched lines) Ig H-chain containing chromosomes in meiosis. Upper pathway generates an LG15-like animal; the lower pathway generates the homozygous LG7 animal. The asterisks (*) represent the point where the prevention of the second polar body extrusion occurs and a viable diploid egg is produced.

animal was easily isolated. Later, it was found to produce both big and small eggs, thus making it clonable.

### Genomic $V_H1$ Gene Sequences

Three genomic libraries representing altogether $1.7 \times 10^6$ recombinants phage (three to four genome equivalents) were made from LG7 red cell genomic DNA and screened unamplified with a *Xenopus* family $V_H1$ cDNA probe at high stringency. A total of twenty eight different $V_H1$ elements were isolated and sequenced, and the alignments of their encoded amino acids are shown in Fig. 3. All exhibit a typical $V_H$ gene structure (Kabat et al., 1991) and all contain specific features characteristic of *Xenopus* $V_H1$ genes. The complementarity-determining regions (CDRs) show limited variability and many different genes contain identical CDR1s and very similar if not identical CDR2s. The CDR2s exhibit the most sequence divergence which is restricted to their amino-terminal ($NH_2$-) portions (Schwager et al., 1988, 1989). Six sets of the genomic $V_H1s$ contain identical CDR1s and three sets contain identical CDR2s (Table 2). The CDR1s are fifteen nucleotides in length and can differ by as much as 60%. Only the nucleotides at positions 100–103, encoding MET 34, never vary. Genomic g44A was the only gene that contained a CDR1 identical to one published previously from a *Xenopus laevis* genome (p LL1.4; Schwager et al., 1989). The largest sequence difference found in the $V_H1$ CDR1s is a change of nine nucleotides (g46C vs. g345A). LG7 $V_H1$ CDR2s vary in length from 48–54 nucleotides (16–18 amino acids) and can differ by as much as 43% (g2A vs. g345A). Overall nucleotide sequence identities range from 82–96%, and as expected, the framework (FR) regions are the most highly conserved. The $V_H1$ nucleotide sequences beginning with the initiation codon (ATG) of the split leader and

TABLE 2
$V_H1$ Genomics That Share CDRs

| CDR1s | CDR2s |
|---|---|
| g7C, g15 | g7B, g7C |
| g4A, g10A | g342A, g342B |
| g35, g2A, g13 | g352, g2B |
| g343, g10B | |
| g342A, g342B | |
| g22, g349B | |

```

                                          CDR1                     CDR2

                                         |   |              |                 |

           ----+----1----+----2----+----3----+----4----+----5----+----6----+----7----+----8----+----9----+----0---

LG7G345A   DVQLAQSEPVVIKPGGSHKLSCTASGFTFSSTWMHWVRQASGKGLQWLCQI-HPDGSSNTYYADSVKGRFTISRDNNNSK*YL*MNNLQTEDTAVYYCA----  M97004

FIGURE 3. Alignments of encoded animo acids of $V_H1$ genomic genes (in one letter code) beginning with residue 1. The $V_H1$ genes in bold type were found expressed in a cDNA library made from immunized LG7 frogs. Sequence identities are indicated by (') and gaps (-) are introduced to maximize homology. The asterisks (*) indicate stop codons. The CDR boundaries are marked and are according to Kabat et al. (1991). Genes g345A and g342A are pseudogenes and gene g5 is truncated due to only being partially sequenced. Master sequence was chosen for length. GenBank accession numbers are at the right margin.

continuing to the conserved nonamer of the recombination signal sequence (RSS) are aligned in Fig. 4. The conserved octamer ATGCAAAT and sequences analogous to a TATA box that are found 5' of all $V_H$ genes (Parslow et al., 1984) and act as the Ig H-chain promoter (Wirth et al., 1987) were identified in twenty five of the $V_H1$ genes. Three of the genes, g349A, g349C, and g343, were cloned using a restriction site just 3' of their leader sequences and so their promoters were not identified. Genes g349A and g343 are both viable $V_H$ genes because they are found expressed in an LG7 cDNA library (Wilson et al., 1992). Very little sequencing was performed 5' of the leader sequences and consequently no second possible promoter regions, as have been previously identified in $V_H1$ genes (Schwager et al., 1989), were found. The LG7 $V_H1$ leader regions range from sixteen to nineteen amino acids in length and several genes (e.g., g7B, g7C, g2A, g35) contain almost identical leader sequences. Gene g44A has the longest leader intervening intron of 108 nucleotides (Fig. 4A). The $V_H1$ genes varied in overall length from 98–101 amino acids. The heptamers of the RSS are identical in all but one of the $V_H1$ genes. Two nucleotide substitutions in g22 change its heptamer to CACA<u>C</u>T<u>A</u>. The nonamer sequences are much more variable and only six genes contain the concensus $^G/_A$CAAAAACA previously identified as the $V_H1$ nonamer (Schwager et al., 1989). The RSS 23-bp spacers vary by a wide range. Six genes contain identical 23-bp spacers, whereas others differ by more than 50% (g345A vs. g352).

Many of the genomic $V_H1$s were isolated more than once from both the same library and from the different libraries. Eight of the recombinant phage contained more than one $V_H1$ gene (e.g., g349A, B, C) and at least four recombinant phage (from seven chosen at random) tested positive for members of additional $V_H$ families by hybridization with family-specific oligonucleotide probes (Haire et al., 1990). Three of the recombinant phage contained $V_H$ II hybridizing fragments and one different recombinant phage contained a $V_H$ VIII hybridizing fragment (data not shown). These results in part agree with recent reports that show evidence for interpersion of *Xenopus* $V_H$ families (Schwager et al., 1989; Haire et al., 1991).

Two of the twenty eight genomic genes are clearly pseudogenes. Gene g345A contains two stop codons in its FR3 region, beginning at nucleotide positions 238 and 247, and gene g242A contains a stop codon in its leader sequence. Genes g342A and 342B are located on the same recombinant phage and differ by four bases. Both genes contain an extra different base in their leader intron sequences and, more important, g342A contains two extra bases in its leader, one of which is responsible for generating the stop codon (TAA) directly after the initiation codon ATG. The close sequence similarities and their close association in the DNA suggest that these two genes are the result of a recent gene-duplication event. Genes g7B and g7C probably also represent another example of recent gene duplication; they are found on the same recombinant phage and differ by thirteen bases in their coding regions. Genes g15 and g7C only differ by one base in their CDR2 regions, and are identical everywhere else from 100 bp 5' of their octamer to 80 bp 3' of their RSS sequences (data not shown). Recombinant phage 15 and 7 are different by RFLP analysis, which suggests that these two sequences represent two genes. However, this evidence, even considered with the low (practically nonexistent) frequency of sequencing error (see Materials and Methods) of our reactions is inconclusive. Both genes g7B and g7C were repeatedly isolated from all libraries, whereas g15 was found only once. Also the EcoR1 fragment containing g15 is approximately equal in size to the EcoR1 fragment containing g7C, and so the difference in the two may well be the result of a sequencing artifact.

### IgM Heavy-Chain Region

The four exons ($C\mu1$ through $C\mu4$) encoding the *gilli* $C\mu$ chain of LG7 are shown in Fig. 5. The coding region encompasses 4163 bp from the first amino acid of $c\mu1$ to the polyadenylation signal. The four exons and their splice sites were identified by comparison with the published *laevis* $\mu$ heavy-chain cDNA sequence (Schwager et al., 1988) and with LG7 $\mu$ cDNA sequences (Wilson et al., 1992). The splice sites obey the traditional rule encountered so far for all Ig genes, the splice junction occurs between the first and second base of the joining amino acid (Brüggerman, 1987). In two places, the exon boundaries of the *gilli* $\mu$ gene differ from the boundaries predicted from the *laevis* $\mu$ cDNA for the $C\mu2$ exon. The LG7 $C\mu2$

exon is two amino acids shorter, ending with a cystein making the C$\mu$3 exon two amino acids longer at its amino-terminal end.

The deduced amino-acid sequence of the *gilli* $\mu$ gene differs at thirty six positions from the published *laevis* $\mu$ cDNA sequence (Schwager et al., 1988). The percent homology of the 452 residues is 79.6% (see Fig. 5); at the DNA level, the homology is 96% (1308/1359 bp identical). This polymorphism, relatively higher at the amino-acid level, is not likely to represent the true allelic polymorphism of *Xenopus* Ig allotypes because *X. laevis* and *X. gilli* are different species even though they can produce fertile offspring. In addition, these substantial differences could explain why species-specific antiheavy-chain

**A**

```

           ----+----1----+----2----+----3----+----4----+----5----+----6----+----7----+----8----+----9----+----0----+----1----+----2

LG7G345A   -ATGAAGTTGTTTCTTGTTATGTT-AAT------GACACTTTTATCAGGTAA----------------TAAAAATAAGGAATAATACAATTACAATTATCACATAACATTTTATTTTTAA

LG7G7B     -''''''''T'''''''''''T''-'T'------'T''''A''G''''''''----------------G''TGG'CTTT'TGG'''TT'GA''TCCA''A'TC'C'T'ACA'T''A''''

LG7G7C     -''''''''''A'''''''''T''-'T'------'T''''A''G''''''''----------------G''TGT'CTTT'TGG'ATATTAG'ACTCC'AT'ATCTCATAACAT'''A'T'

LG7G15     -''''''''''A'''''''''T''-'T'------'T''''A''G''''''''----------------G''TGT'CTTT'TGG'ATATTAG'ACTCC'AT'ATCTCATAACAT'''A'T'

LG7G4A     -'''''''CA''G'''''T'C''-'''TTTCTT''TGTG''''''''''G----------------G''TGT'CTAATTAT''TG'''A'A'GAAT'ACA''C'T'A'A'TC''''T'

LG710A     -''''''A'T'''T'''''G'TA'-'''------'T'''''''G''''''G----------------G''TGT'TTAA'''T''TG'''ACT'GAAT'A'A''''T'A'''TAAAACT'

LG7G349A   -'''''''CA'''A'C'''GTAA-T'ACACT--'T''TA''''''''''''-------------AACTGTC'G'C'TATT'AT''TA'TAATGCT'GTAAGAT'G'TG''A'TAAAACTG

LG7G341    -'''''''''C''''''''TGT''-'''ATTGCTCT''GG''''''''''''----------------G''TGT'T''''GAT'CTG'''A'TT'AAT'ATTCCTCA'GC'ATCAC'''T

LG7G13     -''''''''TC''''''''''C''-'''ATTACTCT''GG''''''''''''----------------G''TGT'C''''GAT'CTG'''A'TT'AAT'ATTCCTCTTGC'ATCAC'''T

LG7G2A     -'''''''CA''GT'''''''C''-'''GTTCCT'T'GTG''''''''''G----------------G''T'T'C'''TGAT''CAG''ACTT'CAT'TCTCA'GACA'CAC'''AAC'

LG7G35     -'''''''CA''A'''''T'C''-'''ATTCCT'T'GTG''''''''''G----------------G''TGT'G'''TGAT''CAG''ACTT'CAT'TCTCA'GACA'CAC'''AAT'

LG7G346B   -''''''''''''''C'''''T''-'T'------'T'G''G'GT''''''''----------------'''TTT''TTT'TGG'GTATTAG'ATTCC'ATCATCTCGTAACG''''''T'

LG7G10B    -'''''''CA''G'''''T'C''-'''GTTCCT'T''TGG''''''''''''----------------G''T'TCCTAATGAT''TG'''A'T'GAAT''TA''C'A'AC''T'''''''

LG7G343    -''''''''''''''CA'''T''-'T'------'T'''''''GT''''''''----------------C''TGT'CTTAT'G'G''TT'GA''TCCA''A'TC''TA'CA''T'A''A''

LG7G4B     -''''''''A'''''''''T'T''-'''------'C''''''''''''''''''---------------TATGTTC'GTAAGGAGTA'T'GAC'ATCAC''''TTAT''T''A'TAAAAACT

LG7G44A    -'''G''''''''''G'''''T''-'''ATTACT'GG''A'''''''''''''GGATGTTATATATATA''T'T'''TAT'TAT'''T'TA''T'TAATAG'AT'CCA'ACA''A'A''T'

LG7G5      -''''''''''''''A'''TGT''-'T'ATTCCTCT''GG''''''''''''----------------G''TGT'CT'''GAT'CTGG''A'ATCCAT''CTCA'GGTA'CAC''A'A'T

LG7G349C   -'''''''''TA''''''''GGTG'-T''ATTCCT'TT'TG''''''''''''----------------G''TGT'C''''GAT''TGT''ACT'CA'T''C''AC'T''CAATG'AA''C

LG7G17B    -''''''''''''G''''''T'CC'-'''ATCCCT'T'''AGC''''''''''---------------TAC'GTTG''''TNAT'''GTTCAGG'GA''CTGG''GCT'AAA'TACA'C'C

LG7G342B   -'''''''CA''G'''C'T''''-'''ATCCCT'T''TGC''''''''''G---------------GG''GTTGT'AAG'''T'GA'TAATAT'A'TC''AT''TA'''''TG''AA''

LG7G342A   ATGT''''CA''G'''C'T''''G'''ATCCCT'T''TGC''''''''''G---------------GG''GTTGT'AAG'''T'GA'TA''T''''CCA'TTAT'AT''''G''AAA''

LG7G22     -''''''''''''G'T''''T'CC'-'''ATCCC'GT''TGGTT''''''''----------------''C'GTA'CA'GTATTATA'T'C'GG'GATA'TG'ATG'TAAAAT'ACA'G'

LG7G46C    -'''''''''AA'''''''''''''-'''------'''''G''''''''''''----------------''''TA'G'A'TA'TACA'T'ACA'T'ATCACATA'CAT'''AT''''A'C

LG7G2B     -'''''''CA''GT'''''''CC'-'''ATCCCT'T''TG''''''''''''----------------''C'GTGTTTATGAG'''TCTGATA'TAA'ATTGC'G'AGACA'CAC'''T'

LG7G21     -''''''''''''''A'''TGT''-'T'GTTCCTCT''GG''''''''''''----------------G''T'T'C'''GAT'CTGG''A'TTC'AT''CTCA'G'TA'CAC''A'A'T

LG7G352    -''''''''''''''''''TAT''-'C'ATTCC'GTTATGG'''''''''''----------------G''TGT'T''''GATG''T'TAACATT'CCCA'G''AT''A'G''A'AAACG

LG7G349B   -''''''''''''''A'''T'T''-'''------''''''''''''G'''''----------------G''TGT''TTT'T'G'GTA'TAG'AT'C'TAA'T''GTTA''CA'AAC'ATC

LG7G331    -''''''''''''''''''T'T''-'T'GTTACT'G''TG''''''''''G----------------G''TGT'CCAC'CAT''TG'''A'TAGCAT'ATAT'T'T''AA'T''A''''

```

```
           ----+----1----+----2----+----3----+----4----+---
LG7G345A   ACAATCGTTATGCCTCTTCTTTTTCC---------AGGAGGTCACTGT
LG7G7B     'A'''ATCAT'ATACTG'A'C'CAT'TTTTCC---'''G'''''''''
LG7G7C     'A''ATA'C''TATA''GTA'C'CATCTTTTCC--'''G'''''''''
LG7G15     'A''ATA'C''TATA''GTA'C'CATCTTTTCC--'''G'''''''''
LG7G4A     'A''A'A'C''TATGTC'''C''TTCC-------'''''T'''''''
LG710A     T'TT'ATG'C'TTT'TCC-----------------'''GT'''''''
LG7G349A   CATT'AT''C'CT''T'AT''C'CT'TTTGTCCT-''''T''''''''
LG7G341    'AT'AAC'GG'TTTATG'''AA'CTATTTC-----'''''T'''''''
LG7G13     'AT'AGCGNG'TTTATG'''GA'CTATTTC-----''''''''''''''
LG7G2A     'TTGCTC''TAT'T'T'C'----------------'''''''''''''
LG7G35     'ATGCTC''TAT'T'T'C'----------------'''''T'''''''
LG7G346B   TT''AAAAATAC'A'TA'G'C''ATGTTTTCC---'''''T'''''''
LG7G10B    CT'TCGT'ATGT'TGA'CTA'''C-----------'''''T'''''''
LG7G343    'A'''AC'ATAATG''''A'G'''TTCC-------'''T'T'''''''
LG7G4B     'TC''T''CTCA'''T'''C---------------'''''T'''''''
LG7G44A    TA''AAAAA'AAAA''A'TA'G'CT''TCTGTTCC''G'T'''T'''
LG7G5      'A'C'GT''T'TATGTC''A'C'ATTTC-------''''''''''''''
LG7G349C   GGTC'TTA'G'CT'ATC'T'CC-------------'''''T'''''''
LG7G17B    TTTTATTAAC'TT''T'ATC'''AA'---------'''G'T'''''''
LG7G342B   ''T'''A'''''TTG'ACT''''C-----------'''''T'T'''''
LG7G342A   CT'TCAT'ATGTTGCA'CT''''C-----------'''''T'T'''''
LG7G22     'TTT'GTAA'CTTTAT''A'C''AAAT--------'''T'T'''''''
LG7G46C    TATCATTA'G'CT''TC'T''CC------------''''''''''''''
LG7G2B     TA''CT'''T'TATCA'''C---------------'''T'T''G''''
LG7G21     G'AC'GT''TCAT'TC'AA'C'A'TTC--------'''G'T'''''C'
LG7G352    GTT''TA'GTCT'A'A''T''CC------------''''T''''''''
LG7G349B   'TGTCTCA'CGTTT'TC------------------'''''T'''''''
LG7G331    'A'C'GA'C''ATT'TCC-----------------'''T---''''''
```

FIGURE 4. Alignment of genomic $V_H$1 genes. (A) Leader sequences beginning at the initiation codon ATG and continuing to the beginning of the $V_H$ region (residue 1). Sequence identities are indicated by (') and gaps (-) are introduced only to conserve length. The stop codon TAA in g342A is underlined. (B) $V_H$1 regions begin at residue 1 and continue through the RSS nonamer. The number of nucleotides from the beginning of the first base of residue 1 is shown above the scale. Gaps are introduced to maximize homology. FR and CDR boundaries are according to Kabat et al. (1991). The stops in g345A are underlined. Master sequence was chosen for length.

FIGURE 4

```
   1 GTAATAAGAATGTCAAAAACCCACAGAGAGCCTGTGAAGATTTGTTCTTTGCACTAACATCAAAACTAACCTTAACTCTTCCACAAACCTGGTTCCAGTGAAATTCC   107

                                                                                                       Ala Thr Ser

 108 TACATTTCATCTAACCACCTTGTAATCATTTACCATCTCTAATCCTAACAACAAATACTCATTCCTTCCCCCTTCTCTTTTGCTTGTATTCTCAG   CT ACT TCA   210

     Lys Ser                 10                  15                  20                  25                  30

     Asn Pro Pro Ser Leu Phe Pro Leu Ile Ser Cys Gly Glu Ser Met Asp Pro Val Thr Ile Gly Cys Leu Ala Lys Asp Phe

 211 AAC CCC CCA TCC CTT TTT CCA CTC ATT TCT TGT GGG GAG TCT ATG GAC CCA GTC ACC ATT GGT TGT TTG GCC AAA GAT TTC   291

                 35          Thr  40                 45                  50                  55

     Leu Pro Glu Thr Ile Ser Phe Ser Trp Gly Asp Lys Asn Asn Ala Ser Tyr Ser Thr Gly Leu Lys Ser Tyr Lys Pro Val

 292 CTC CCT GAA ACT ATT AGC TTC AGC TGG GGA GAT AAG AAC AAT GCC AGT TAC TCC ACT GGC CTC AAG AGC TAT AAA CCT GTG   372

             60                  65                  70                  75              Asn Ile     Gln

     Met Gln Ser Ser Gly Thr Tyr Ser Ala Ser Ser Gln Val Asn Val Ala Ser Ala Val Trp Asp Lys Ser Glu Pro Phe Tyr

 373 ATG CAA TCT TCC GGC ACC TAC TCA GCC AGC TCC CAA GTC AAC GTT GCC TCT GCA GTC TGG GAC AAA AGT GAA CCA TTT TAC   453

      85                 90 Asp Thr Ile     95         Leu     100 Asp Pro

     Cys Asn Ala Lys His Leu Glu Ile Thr Lys Ser Val Glu Val Lys Lys Gly Thr V

 454 TGC AAT GCC AAA CAC CTG GAG ATC ACT AAG AGT GTG GAG GTC AAG AAA GGT ACA G GTAACCTTATTTGAACTTTAAATTCACATAGA   541

 542 AATATATTATCTTGCTCTCTATATTTATTGCCATGAAACATATTTACTTATTGATAAACAGCTCCAAGAATCAACACCACTGATTCAATACAAGTAAGATTATTACT   648

 649 CAGTGATTCTGTCGGAACCACTTTATATTGAGCACAAATAATAAATATAATTTCTTATAGAAGTAATAAAAACATAATTTCTTATATACCTATAATATATATATTTT   755

 756 TTTAATAGAGATGCTGCAAGTGGAATGCATAACTGCATACATATCAAGCAGCCATGCATGAAATATGTGTATTCAATAATTGTCCTCTTTTAAAGGGGTGATATGGC   862

 863 AATCCTAATGAGAACTATACTTTGGTATGCATCTAATACAATAGGCTGAACGCACAAACCCCTACCAAACGATCATATTTCTCTAGGTATTAGTAAACAACTAAAAT   969

 970 GAAAACTTCAGGTGATTAAAATTAATGCTAAATAATTAATGTGTGTTGTTAGTAGTTTCTAGAAGTTAAAATGTTTAATTTCCACATAAGGCTTGTGTCACAATTAA  1076

1077 GGTTATTAGCAGCAACTTTACAACTGTCTGCAAATACAGGTATGGATCTGTTCTTGGAATCTTGGGTTTTTCTGTTTTTTCATTATTTGGAGAACCATGACTTTAGA  1183

1184 TACTAAAATATGTGAACATAAGAAAAAAACTGGGTATGTCATCAGAATGGTATCTCAAATTACAGGGTAGTGTCTAGTTATTACATAGAAAAAAGCAAACCCATAGC  1290

1291 AAGAGAAGAAATGCTTGTTCAAATAGGACTCTATGGGAAATGACAAAAGGTAATTTAAAGGTGAACTACCCCTTTAATTCTTTCCAATTGGCATAATAATCTACTTG  1397

1398 CTTTAAAGAAAACAAAGGAACCTTTTTGGAGCCAGTTTCAAGTAGCAGCTACTTTCCCTGTGTGACATAAGCCTGACTGTGTAAATTATCCTCTTTGCATTCATAAA  1504

1505 TTAAAACTAATAAAACTAATAGAAGTTACATGTAGTACACAACAGAATGTGAACACTAGAAATGATATTTCCTTCCAATATTTTTAGAAAAATCTACTGGTTTATAT  1611

1612 ACAAATGTGACAAAAAACACTGAACTCTTTTTATTATCGGTATTCAGTATACACTGAGGGACAGATTTATTATGTGGTGTTAAAAATGGTGGAATAATACACCACAC  1718

1719 GTCTCCAGCATTGCCGTATAAGAATGGTGTTATTTTTTTTACGCACTTTTTCTTTGACCAACTACTGGCAGAACTTACTTATAAAGGTCTGAATCGATGTGAAATAG  1825

1826 CATTCACATGGCATAAAATAAAACACACCTTGATAAATTCGCCATTTTTACATGGCTTTCTACACCGTTTTTTTTGCCAAATTTCGTTTTACACAGCATAACTATGC  1932

1933 CCCTGAATGTACATATTGCATACAAAAATACAACTGAAGAATGATACATAAAATGAATAGGCCCTGCCCAAAAGAGTTTACGGAGTGACCAGCTTGTAGAGTTGACA  2039

2040 TAAATTATTTTACCCTTTTTTTGTTTATTTGTATCAACACTCACTTTTCTTTAATTAAAGGAGTAACACATCATTAGCTTGGAAGTATACCGGAAATGCGTCTCTGT  2146

                                                              Lys Pro                 110                 115
                                                           al Asn Lys Val Glu Lys Pro Val Val Ser Ile His Pro Pro
2147 TTATCTTATAGGATCGCCTAATCCTATTCTTTTCTATCCCTTTTATCAG     TG AAT AAA GTT GAA AAA CCA GTT GTG TCT ATT CAC CCT CCA  2236

                 120                 125                 130                 135     Thr     Thr His
     Ser Lys Asp Ala Leu Ala Leu Asn Glu Ser Leu Phe Ile Val Cys Leu Ala Thr Asn Phe Asn Pro Lys Asn Ile Val Ile
2237 TCC AAG GAT GCT CTT GCC TTG AAT GAA AGC CTC TTT ATC GTA TGT CTT GCA ACG AAT TTT AAT CCC AAA AAT ATA GTA ATT  2317

         145                 150         Thr 155                 160                 165     Arg         170
     Lys Trp Leu Lys Asn Gly Asn Gln Thr Lys Glu Gly Val Arg Val Glu Glu Pro Val Glu Asp Lys Lys Gly Gly Tyr Glu
2318 AAA TGG CTA AAG AAT GGG AAC CAG ACA AAA GAA GGT GTG AGA GTT GAA GAA CCT GTT GAA GAC AAA AAG GGA GGA TAT GAG  2398

                     175                 180                 185                 190                 195 Glu
     Thr Thr Ser Tyr Leu Ser Ile Thr Arg Lys Glu Trp Asp Leu Asp Thr Leu Tyr Ser Cys Val Val Glu His Ala Gly Ser
2399 ACA ACA TCC TAT CTC TCC ATA ACT AGA AAG GAA TGG GAT TTG GAT ACT TTG TAC TCA TGT GTA GTT GAA CAT GCA GGA TCG  2479

     Ala     200                 205                 210

     Gly Ser Leu Gln Glu Lys Asn Met Ser Lys Ser Leu Met Cys A

2480 GGT TCC TTA CAA GAG AAG AAT ATG AGC AAA TCA CTA ATG TGT G GTAAGTTCTTGTGATGTGATGCTTATTGTTCTACAGATAAGGATCAGTT  2571

2572 TTACTTTAAGAAATTAATTTATGTATAAGTGTTAATACTGTGGGTCATATTTGCTTCAATGCATGTTGGTACATATATAGTTACATATAGTCTTTATCTGATTATGA  2678

2679 CCAATAAAAGCCAAAATATACCAATTTTGAAAGGTATTTTTTGCCTGTAAACAGTGCTAGGAACCGGCACTCACCTCTCCTTAGTACTAATAGCCAGGTATATATAT  2785

2786 ATGAGAGAGATGTTCAGGGTATTTTTTGATGTCATAGTGTTATGCACTACTATTTAAAGGGTTGTGGTCCCTGTGAATTTCAGGTCAGTGAATGTGAAACAGCATCT  2892

2893 CGTTTTCTATTAAAACTGTCTTATGGGGCTAAACATGAAGTGATGATCGGTTATTTTGGTTGAACAAAGCCAAGTAGACTGGCATGTTTGCACAATGTAAGGGTGGC  2999

3000 TTAATAAATGTTTTGTTATTGGAGGGAATTTAACATAAATAATGTATAATTTGCAGTATGGTATCTCACTATTTTTTTTTCCTTTTTTTTCCTTTTTTTTTTCCTTT  3106
```

FIGURE 5

Mabs (like 14G1, see the foregoing) can be produced.

Only 650 bp beyond $C\mu4$ were sequenced and no transmembrane exons were found in that stretch. The segments encoding the *Xenopus* transmembrane region have been published previously (Du Pasquier and Schwager, 1989) and a complete map of the *Xenopus* IgH locus will be published elsewhere (Du Pasquier, in preparation). The *gilli* $\mu$ locus is also characterized by a long intron between $C\mu1$ and $C\mu2$ (1618 bp). This intron contains sequences similar to some enhancer motifs described for mammalian Igs (Staudt and Lenardo, 1991). An octamer and sequences analogous to $\mu$e4 and $\mu$e5 are identified in Fig. 5.

```
                          Pro 215                 220                 225                 230                 235
                   sp Thr His Ile Thr Pro Thr Ser Ile Gln Val Ile Thr Ile Pro Pro Ser Leu Glu Ser Ile Phe Glu Lys
3107  TTCCTCAG     AC ACC CAT ATA ACA CCC ACT AGT ATC CAA GTA ATC ACT ATT CCA CCA TCA TTA GAG AGC ATT TTT GAG AAA    3185

                      240                 245                 250                 255             Val 260
      Lys Ser Ala Thr Leu Thr Cys Leu Val Ser Asn Met Ala Asn Ser Glu Asp Leu Arg Ser Ile Ser Trp Phe Lys Lys Ser
3186  AAA TCT GCC ACA CTT ACC TGC CTG GTC AGT AAT ATG GCT AAC TCT GAG GAT TTG AGA TCA ATA TCC TGG TTT AAA AAA TCT    3266

              265                 270                 275                 280 Tyr         Phe 285
      Gly Thr Gln Glu Ile Pro Leu Lys Thr Glu Leu Gly Asp Ala Ile Tyr Asn Asp Asn Arg Thr Tyr Ser Val Lys Gly Thr
3267  GGT ACT CAA GAG ATA CCA TTG AAA ACA GAA CTG GGA GAT GCA ATC TAT AAC GAT AAC CGC ACC TAT TCT GTA AAA GGA ACC    3347

      290                 Glu                 300                 305                 310             Met 315
      Thr Thr Val Cys Ala Asp Glu Trp Asn Asn Asp Lys Phe Val Cys Lys Val Glu His Thr Glu Leu Ala Ser Val Lys Glu
3348  ACC ACT GTC TGC GCT GAT GAA TGG AAT AAC GAC AAG TTT GTC TGC AAA GTG GAA CAC ACA GAG CTG GCT TCA GTG AAG GAG    3428

          Leu Pro Leu
      Val Phe Leu Phe Lys Glu Lys G
3429  GTC TTT CTC TTT AAA GAA AAA G GTAAGGCCATCTACCTGTACTGCACAGTACTAATCAGACAGAACAACTATAATAAAATAGCCATTATACTTGATACC    3527

3528  AATATAGCACACATTTTTCATGTGCAGAGGTTGTTATTCATCCACTGATCTGATAGCTATTATTAATATACAATTAATAAATTTCTATTAGGGTCTGTTTTAAGCAC    3634

3635  CAAAGACCAACATTCTGTAGAAAGTATAATTTTCTACAATAGAGTAGATCATCAACCTGTAAAATAAATTGTGACATCCATTCGAGTTGTATGTTAAGGGTGTTTTT    3741

                                                                                                  325
                                                                                               ly Glu Tyr Asn Thr
3742  ACTGATATCTGTAGAGGAAGCATTTGTGTGAGTAATCCCTTCAAAAACTTTGAGATTTGTAACTCCTAATTTTCTATTTTAAAACAG  GA GAA TAC AAT ACA    3842

          330         Phe Ser 335                 340                 345                 350                 355
      Pro Ser Val Tyr Val Phe Pro Pro Pro Leu Glu Glu Leu Ser Lys Arg Glu Thr Ala Thr Leu Thr Cys Leu Val Lys Gly
3843  CCA TCT GTT TAT GTT TTC CCA CCA CCT CTT GAG GAA TTG TCT AAG AGA GAA ACT GCC ACC TTG ACA TGC TTG GTT AAA GGG    3923

                      360                 365         Lys     370                 375                 380
      Phe Ser Pro Ser Glu Ile Phe Val Lys Trp Leu His Asn Asn Glu Ala Val Pro Lys Gln Asn Tyr Ile Asn Thr Ser Ile
3924  TTC AGC CCC TCT GAA ATA TTT GTA AAA TGG CTT CAC AAC AAT GAG GCG GTT CCA AAA CAA AAT TAC ATA AAT ACC AGC ATC    4004

              385     Leu         390                 395                 400     Asn         405 Ile
      Asn Asp Glu Leu Tyr Pro Lys Gly Gln Lys Ser Gly Lys Phe Phe Leu Tyr Ser Leu His Thr Ile Asp Phe Lys Asp Trp
4005  AAT GAC GAG CTT TAT CCC AAA GGA CAG AAG AGT GGA AAG TTC TTT CTG TAC AGT CTT CAC ACC ATA GAC TTT AAA GAC TGG    4085

      410                 415                 420                 425                 430                 435
      Asp Ala Gly Asp Ser Phe Ser Cys Val Val Gly His Glu Ser Leu Pro Leu Gln Leu Thr Gln Arg Ser Ile Asp Lys Ser
4086  GAT GCT GGT GAT AGT TTT TCC TGT GTG GTT GGC CAT GAG TCA TTA CCA CTT CAG CTG ACC CAA AGG AGC ATT GAC AAG TCT    4166

                  440                 445                 450

      Ser Gly Lys Pro Thr Asn Val Asn Val Ser Leu Val Leu Ser Asp Thr Cys  *   *

4167  TCT GGT AAA CCT ACT AAC GTG AAT GTG TCC CTC GTC TTG TCT GAT ACC TGT TAGTGATCATCTCCAAAGACCCTATAACTCCATCTCATG    4256

4257  TTCTTCCCTATATGGTGTAATGGACAAGGCGGAGGGAGAAGTGTTGACTTTATGTTTGTCTGTCTGTTCTTGTACATTTTTTGTCTGCAAAATTAAAAGTAGAAATA    4363

4364  AAAATCAATTTATACAATGTTTGTTTGTATGCATGCAATTTGGGAAAGAAGTGGTGATTTGGAAAATTGAAATACTTCACCACTGGTTTGTAGCCTGCATTAGGGAT    4470

4471  TTGAATTCCAGATACACCTACACCTTTTCATAAAACCCACAGCCATTAGCTTCAGTAGGAATCCAAAAGCTCAGAGCTGCATAGGGTAAGAACTTAACAAAGGTCAC    4577

4578  ATTACTACTTGATTAGTTTCATTTATAAAGAAATTACATATGACTAGGAAAGGGAATGCTTACTAGCCAAGATGTCCAAATAAACATCATTCAGTTAATTTGAAAGT    4684

4685  GTTTCATGTGCTTTCTATGTTATAGGCAATGTGTGTTCAACTTAGAACATATGAGACAAAACAAACTGTTATGGGCATATTTTTCAAAGTGTGAAATTACTCCCACT    4791

4792  TTCAATTCTTTTCTACTGGTTTTTGTTGTACACTACAAG                                                                        4830
```

FIGURE 5. Nucleotide and inferred amino-acid sequences of the LG7 IgM heavy-chain gene. The inferred amino acids are indicated above the nucleotides. Only the amino acids of *laevis* $\mu$ (from the cDNA sequence; Schwager et al., 1988) that differ from *gilli* $\mu$ are shown. These residues are shown above the LG7 *gilli*-encoded amino acids Sequence motifs in the CH1 to CH2 intron that are similar to Ig-enhancer seuqencers are underlined. These include a SITE E, E-boxes ($\mu$e5, $\mu$e3, $\mu$e4), and a $\mu$B motif. A sequence identical to the KBF-A motif in the kappa-chain enhancer is underlined and overlined. The octamer sequence is double underlined. Ig-enhancer sequences are from Staudt and Lenardo (1991). Polyadenylation sites are in bold. GenBank accession number of *gilli* $\mu$ is M97008.

### DISCUSSION

In this study, we examined a clone of isogenic *Xenopus*, the *X. laevis*/*X. gilli* hybrid LG7, which probably developed accidentally from a LG15 small egg (Kobel and Du Pasquier, 1986) with a recombined genome. Because this clone is homozygous at the heavy-chain locus, its genetic simplicity makes it the best available strain for comparing germ line with cDNA Ig sequences. These comparisons are necessary to detect the presence of somatic mutations, an issue that has remained unanswered in the lower vertebrates for the past 18 years, ever since the major differences in antibody repertoires were first reported between amphibians and mammals.

In addition to establishing a dictionary of $V_H1$ sequences from LG7, we have sequenced the $\mu$-chain gene. This will be useful for future studies with conventional heterozygous LG hybrids in order to identify origins of $\mu$ cDNAs.

All of the twenty eight $V_H1$ genes sequenced exhibited the typical vertebrate $V_H$-gene structure (Kabat et al., 1991) and sequence identities ranged from 82–96%, thus easily fulfilling the criteria of $V_H$-gene family membership (Brodeur and Riblet, 1984). In addition, all of the $V_H1$s contain specific features and conserved nucleotides previously described for *Xenopus* $V_H1$ genes (Schwager et al., 1989). Many shared CDR1s and CDR2s are similar even though by overall comparison they show the most sequence divergence. Most of this variability is in the first eight to ten amino acids of the CDR2s. The LG7 $V_H1$ sequences further reinforce, by allowing for greater comparisons, the hypothesis that the low heterogeneity of the *Xenopus* $V_H$ pool (at least for this family) may be the result of recent expansion events (Schwager et al., 1989; Du Pasquier and Schwager, 1991).

We are aware that the screening process may have missed genes and that calculating $V_H$ family size by Southern blotting can be inaccurate because restriction fragments may contain more than one gene or may comigrate in the gel. The size of the LG7 $V_H1$ family was originally estimated to contain at a maximum thirty genes and Southern blot analyses with frequent six or even four base cutters are consistent with this estimate (data not shown). Because studies in outbred *laevis* and other LG hybrids place the $V_H1$ family size at approximately sixty in heterozygous individuals (Schwager et al., 1989; Haire et al., 1990), we believe that very few LG7 genes are missing. Indeed, when the twenty eight germ-line genes sequenced in this study were used to analyze LG7 somatic mutants (Wilson et al., 1992), only four out of fifty five cDNAs could not be unambiguously assigned to one of these germ-line $V_H$s.

The LG7 *gilli* $C\mu$ segment conforms to the organizational pattern seen in all other vertebrate $C\mu$ loci. There are four exons that encode the four protein domains of the secreted $\mu$ heavy chain and a detailed analysis of the inferred amino acid identities between *Xenopus* $\mu$ chain and those of other vertebrates can be found in Schwager et al. (1988). One unusual feature of the *gilli* $\mu$ gene is the presence of enhancer motifs in the $C\mu1$ to $C\mu2$ intron. Whether these sequences have any enhancer function remains to be investigated. Enhancer like sequences are also found 5' of the putative switch region of the *Xenopus laevis* IgM gene (Du Pasquier, unpublished), a location analogus to the site of the mammalian IgH-chain enhancer. The $C\mu1$, $C\mu2$ intron length, however, is not unusual. Introns of similar lengths are found between $C\mu1$ and $C\mu2$ of the channel catfish, *Ictalurus punctatus*, and between $C\mu2$ and $C\mu$ 3 of the horned shark, *Heterodontus francisci* (Wilson et al., 1990; Kokubu et al., 1988).

The LG7 hybrids are the first *Xenopus* clones to be homozygous at their IgH locus and preliminary evidence indicates that they may well be homozygous for the $\rho$ light-chain locus. Except for the MHC locus, which is heterozygous (Bernard et al., 1979), homozygosity at other LG7 genetic loci has yet to be investigated. In the future, it may well prove to be worthwhile to create other clonable homozygous LG hybrids by using pressure or cold-temperature shock to increase the frequency with which the second meiotic division does not occur. This technique was previously exploited to study histocompatibility antigens (Kobel and Du Pasquier, 1977).

### MATERIALS AND METHODS

### Genomic DNA Preparation and Library Construction

High-molecular-weight DNA was prepared from *Xenopus* LG erythrocytes lysed in TES buffer (10 mM Tris, pH 8.0, 10 mM EDTA, 400 mM

NaCl, 0.2% SDS), containing 100 $\mu$g/ml proteinase K. LG7 small-egg progeny DNA was prepared in an identical manner except that tissues from 3-day-old tadpoles were homogenized in TES buffer. DNA was extracted as previously described (Kiefer, 1990).

LG7 genomic libraries were made from DNA partially digested with Sau3A or Mbo1 and size fractionated by sucrose gradient centrifugation (Davis et al., 1986). Fragments of approximately 20 kb were packaged into EMBL3 vectors (Stratagene) following manufacturers' recommended protocol. The libraries contained approximately $3.6 \times 10^5$ (Sau3A) and $5.6 \times 10^5$ (Sau3A) and $8 \times 10^5$ (Mbo1) recombinants. All libraries were screened unamplified with $V_H1$ or $C\mu$ probes, as previously described (Wilson et al., 1986). Identical $V_H1$ genes were isolated from all three libraries. The $C\mu$ genes were isolated and sequenced from the library Sau3A 3.

### Southern Blot Analysis

Genomic DNA (5–10 $\mu$g) was digested to completion with restriction enzymes, electrophoresed on 0.7% agarose gels, transferred to nitrocellulose or nytran filters (Schleicher and Schuell) and hybridized (Schwager et al., 1991). All probes were labeled by the method of Feinberg and Vogelstein (1983). After overnight hybridization, the Southern blots were washed at high stringency (65°C, 0.1×SSC, 0.1% SDS). X-ray film exposure was at –80°C. The DNA probes for $V_H1$, $V\rho$, $C\rho$ were kindly provided by our colleague, J. Schwager.

### Subcloning and Sequencing

Fragments containing $V_H1$ genes were subcloned into Bluescript plasmid vectors (Stratagene) and sequenced on both strands by primer extension using the dideoxynucleotide triphosphate chain termination reaction method (Sanger et al., 1977). Synthetic oligonucleotides were prepared by H. R. Kiefer's laboratory (Basel Institute for Immunology). The error frequency of the modified T7 polymerase (USB) used in our sequencing reactions must be very low because two independent genomic $C\mu$ clones were sequenced (4830 bp each) with no base differences being found.

### Computer Analysis

DNA sequences were aligned in pairs with a program based on the algorithm of Needleman and Wuntsch (1970). Each sequence was aligned to a "master" and their pairwise alignments were used as input for a multiple alignment program based on a heuristic algorithm. All computer programs were written by Charles Steinberg (Basel Institute for Immunology).

### Enzyme-Linked Immunoabsorbent Assay and Chromosome Analysis

Serum was obtained from LG7, LG15, and outbred *laevis* frogs (two each) and diluted 1/10 in amphibian PBS (standard PBS diluted 1.3 fold). Microtitre plate wells were coated with 50 $\mu$l of diluted frog serum overnight at 4°C. After washing the plates, assays were performed using the $\beta$-Galactosidase Hybridoma Screening Kit (BRL) following the manufacturer's recommended procedure. The primary antibodies were Mabs 14G1, 10A9, 409B8, and 11D5 (Du Pasquier et al., 1985).

Chromosome spreads were prepared using a procedure previously described (Du Pasquier et al., 1985).

### ACKNOWLEDGMENTS

We thank Drs. Charles Steinberg and Jacques Robert for critical reading of the manuscript. The Basel Institute for Immunology was founded and is supported by F. Hoffmann-La Roche Ltd., Basel, Switzerland.

*(Received June 27, 1992)*

*(Accepted July 10, 1992)*

### REFERENCES

Bernard C.C.A., Bordmann G., Blomberg B., and Du Pasquier L. (1979). Immunogenetic studies on the cell-mediated cytotoxicity in the clawed toad *Xenopus laevis*. Immunogenetics **9:** 443–459.

Brandt D.C., Griessen M., Du Pasquier L., and Jaton J.C. (1980). Antibody diversity in amphibians: Evidence for the inheritance of idiotypic specificities in isogenic *Xenopus*. Eur. J. Immunol. **10:** 731–736.

Brodeur P.H., and Riblet R. (1984). The immunoglobulin heavy chain variable region (Igh-V) locus in the mouse. I. One hundred Igh-V genes comprise seven families of homologous genes. Eur. J. Immunol. **14:** 922–930.

Brüggerman M. (1987). Genes encoding the immunoglobulin constant regions. *In:* Molecular genetics of immunoglobulin, Calabi F., and Neuberger M.S., Eds. (Amsterdam: Elsevier Science Publishers), pp. 51–79.

Davis L.G., Dibner M.D. and Battey J.F. (1986). Basic methods in molecular biology (New York: Elsevier), pp. 171–174.

Du Pasquier L., Flajnik M.F., Guiet C., and Hsu E. (1985). Methods used to study the immune system of *Xenopus* (Amphibian, Anura). *In:* Immunological methods III, Lefkovits I., and Pernis B., Eds. (London: Academic Press), pp. 425–465.

Du Pasquier L., and Schwager J. (1989). Evolutions of the immune system. *In:* Progress in immunology VII, Melchers F., Ed. (New York: Springer Verlag), pp. 1246–1255.

Du Pasquier L., and Schwager J. (1991). Immunoglobulin genes and B cell development in amphibians. *In:* Mechanisms of lymphocyte activation and immune regulation III, Gupta S., Ed. (New York: Plenum Press), pp. 1–9.

Du Pasquier L., and Wabl M.R. (1978). Antibody diversity in amphibians: Inheritance of isoelectric focusing antibody patterns in isogenic frogs. Eur. J. Immunol. **8:** 428–433.

Feinberg A.P., and Vogelstein B. (1983). A technique for radiolabelling DNA restriction fragments to high specific activity. Anal. Biochem. **132:** 6–13.

Haire R.N., Amemiya C.T., Suzuki D., and Litman G.W. (1990). Eleven distinct $V_H$ gene families and additional patterns of sequence variations suggest a high degree of immunoglobulin gene complexity in lower vertebrates, *Xenopus laevis*. J. Exp. Med. **171:** 1721–1737.

Haire R.N., Ohta Y., Litman R.T., Amemiya C.T., and Litman G.W. (1991). The genomic organization of immunoglobulin $V_H$ genes in *Xenopus laevis* shows evidence for interspersion of families. Nucleic Acids Res. **19:** 3061–3066.

Kabat E.A., Wu T.T., Reid-Miller M., Perry H.M., and Gottesman K.S. (1991). Sequences of proteins of immunological interest (Bethesda, MD: National Institutes of Health).

Kiefer H.R. (1990). United States patent no. 4,946,952.

Kobel H.R., and Du Pasquier L. (1975). Production of large clones of histocompatible, fully identical clawed toads *(Xenopus)*. Immunogenetics **2:** 87–91.

Kobel H.R., and Du Pasquier L. (1977). Strains and species of Xenopus for immunological research. *In:* Developmental immunobiology, Solómon J.B., and Horton J.D., Eds. (Amsterdam: North Holland-Elsevier), pp. 299–306.

Kobel H.R., and Du Pasquier L. (1986). Genetics of polyploid *Xenopus*. Trends Genet. **2:** 310–315.

Kokubu F., Hinds K., Litman R., Shamblott M.J., and Litman G.W. (1988). Complete structure and organization of immunoglobulin heavy chain constant region genes in a phylogenetically primitive vertebrate. EMBO J. **7:** 1979–1988.

Nace G.W., Richards C.M., and Asher J.H., Jr. (1970). Pathenogenesis and genetic variability. I. Linkage and inbreeding estimations in the frog, *Rana pipiens*. Genetics **66:** 349–368.

Needleman S.B., and Wunsch C.D. (1970). A general method applicable to the search for similarities in the amino acid sequence of two proteins. J. Mol. Biol. **48:** 443–453.

Parslow T.G., Blair D.L., Murphy W.J., and Granner D.K. (1984). Structure of the 5' ends of immunoglobulin genes: A novel conserved sequence. Proc. Natl. Acad. Sci. USA **81:** 2650–2654.

Sanger F., Nicklen S., and Coulson A.R. (1977). DNA sequencing with chain-terminating inhibitors. Proc. Natl. Acad. Sci. USA **74:** 5463–5467.

Schwager J., Bürckert N., Courtet M., and Du Pasquier L. (1989). Genetic basis of the antibody repertoire in *Xenopus:* Analysis of the $V_H$ diversity. EMBO J. **8:** 2489–3001.

Schwager J., Bürckert N., Schwager M., and Wilson M. (1991). Evolution of immunoglobulin light chain genes: Analysis of *Xenopus* IgL isotypes and their contribution to antibody diversity. EMBO J. **10:** 505–511.

Schwager J., Mikoryak C.A., and Steiner L.A. (1988). Amino acid sequence of heavy chain from *Xenopus laevis* IgM deduced from cDNA sequence: Implications for evolution of immunoglobulin domains. Proc. Natl. Acad. Sci. USA **85:** 2245–2249.

Staudt L.M., and Lenardo M.J. (1991). Immunoglobulin gene transcription. *In:* Annual review of immunology, Paul W.E., Fathman C.G., and Germain R., Eds. (Palo Alto: Annual Reviews Inc.), pp. 373–398.

Wabl M.R., and Du Pasquier L. (1976). Antibody patterns in genetically identical frogs. Nature **264:** 642–644.

Wirth T., Standt L., and Baltimore D. (1987). An octamer oligonucleotide upstream of a TATA protif is sufficient for lymploid-specific promoter activity. Nature **329:** 174–178.

Wilson M., Middleton D., Alford C., Sullivan J.Y., Litman G.W., and Warr G.W. (1986). Putative immunoglobulin $V_H$ genes of the goldfish, *Carassius auratus*, detected by heterologous cross-hybridization with a murine $V_H$ probe. Vet. Immunol. Immunopath. **12:** 21–28.

Wilson M., Hsu E., Marcuz A., Courtet M., Du Pasquier L., and Steinberg C.M. (1992). What limits affinity maturation of antibodies in *Xenopus*-the rate of somatic mutation or the ability to select mutants? EMBO J. **12:** in press.

Wilson M.R., Marcuz A., van Ginkel F., Miller N.W., Clem L.W., and Warr G.W. (1990). The immunoglobulin $\mu$ heavy chain constant region gene of the channel catfish, *Ictalurus punctatus:* An unusual mRNA splice pattern produces the membrane form of the molecule. Nucleic Acids Res. **18:** 5227–5233.

